# Strategies for Enriching Variant Coverage in Candidate Disease Loci on a Multiethnic Genotyping Array

**DOI:** 10.1371/journal.pone.0167758

**Published:** 2016-12-14

**Authors:** Stephanie A. Bien, Genevieve L. Wojcik, Niha Zubair, Christopher R. Gignoux, Alicia R. Martin, Jonathan M. Kocarnik, Lisa W. Martin, Steven Buyske, Jeffrey Haessler, Ryan W. Walker, Iona Cheng, Mariaelisa Graff, Lucy Xia, Nora Franceschini, Tara Matise, Regina James, Lucia Hindorff, Loic Le Marchand, Kari E. North, Christopher A. Haiman, Ulrike Peters, Ruth J. F. Loos, Charles L. Kooperberg, Carlos D. Bustamante, Eimear E. Kenny, Christopher S. Carlson, on behalf of PAGE Study

**Affiliations:** 1 Division of Public Health Sciences, Fred Hutchinson Cancer Research Center, Seattle, Washington, United States of America; 2 Department of Genetics, Stanford University, Stanford, California, United States of America; 3 Division of Cardiology, George Washington University School of Medicine and Health Sciences, Washington, DC, United States of America; 4 Department of Genetics, School of Arts and Sciences, Rutgers University, Piscataway, New Jersey, United States of America; 5 The Charles Bronfman Institute for Personalized Medicine, The Icahn School of Medicine at Mount Sinai, New York, New York, United States of America; 6 The Department of Preventive Medicine, The Icahn School of Medicine at Mount Sinai, New York, New York, United States of America; 7 Cancer Prevention Institute of California, Fremont, California, United States of America; 8 Department of Epidemiology, Gillings School of Global Public Health, University of North Carolina, Chapel Hill, North Carolina, United States of America; 9 Department of Preventive Medicine, Keck School of Medicine, University of Southern California/Norris Comprehensive Cancer Center, Los Angeles, California, United States of America; 10 Division of Intramural Research, National Institute of Child Health and Human Development, National Institutes of Health, Bethesda, Maryland, United States of America; 11 Division of Genomic Medicine, National Human Genome Research Institute, National Institutes of Health, Bethesda, Maryland, United States of America; 12 Department of Epidemiology Program, University of Hawai’i Cancer Center, Honolulu, Hawai’i, United States of America; 13 Department of Epidemiology, University of Washington, Seattle, Washington, United States of America; Ohio State University Wexner Medical Center, UNITED STATES

## Abstract

Investigating genetic architecture of complex traits in ancestrally diverse populations is imperative to understand the etiology of disease. However, the current paucity of genetic research in people of African and Latin American ancestry, Hispanic and indigenous peoples in the United States is likely to exacerbate existing health disparities for many common diseases. The Population Architecture using Genomics and Epidemiology, Phase II (PAGE II), Study was initiated in 2013 by the National Human Genome Research Institute to expand our understanding of complex trait loci in ethnically diverse and well characterized study populations. To meet this goal, the Multi-Ethnic Genotyping Array (MEGA) was designed to substantially improve fine-mapping and functional discovery by increasing variant coverage across multiple ethnicities at known loci for metabolic, cardiovascular, renal, inflammatory, anthropometric, and a variety of lifestyle traits. Studying the frequency distribution of clinically relevant mutations, putative risk alleles, and known functional variants across multiple populations will provide important insight into the genetic architecture of complex diseases and facilitate the discovery of novel, sometimes population-specific, disease associations. DNA samples from 51,650 self-identified African ancestry (17,328), Hispanic/Latino (22,379), Asian/Pacific Islander (8,640), and American Indian (653) and an additional 2,650 participants of either South Asian or European ancestry, and other reference panels have been genotyped on MEGA by PAGE II. MEGA was designed as a new resource for studying ancestrally diverse populations. Here, we describe the methodology for selecting trait-specific content for use in multi-ethnic populations and how enriching MEGA for this content may contribute to deeper biological understanding of the genetic etiology of complex disease.

## Introduction

Over the last decade genetic research has made marked advances in cataloging variants associated with complex traits and human diseases, which in turn has shed new light on etiological processes. Initial GWAS prioritized genetically homogeneous populations to help prevent spurious findings that could result from population stratification. As such, initial genotyping platforms were intended to capture common genetic variation in populations of European descent, and later designed to enable efficient imputation of variant reference panels derived from very large sets of European-descent controls [1]. The sparsity of genetic research in American minority populations has led to insufficient sample sizes and inadequate genomic tools for diverse populations. Limited understanding of the genetic heterogeneity in disease loci across populations could greatly limit variant discovery efforts and exacerbate existing health disparities in many complex diseases [2]. For example, development of genetic risk models based on European-descent genetic architecture may, in some cases, have reduced predictive accuracy in populations with greater genetic diversity and less linkage disequilibrium (LD) among variants such as African-descent populations [3]. Furthermore, in conjunction with decreases in genotyping cost, enriching arrays for functional variation and inclusion of more variants in known GWAS regions can be used as an advantageous research tool. By leveraging differences in genetic architecture across ancestral populations, transethnic studies can be used to hone in on likely causal variants in known disease or trait loci. This insight can enable rich inferences about the underlying biology of complex diseases and may improve risk modeling across diverse populations.

The Population Architecture using Genomics and Epidemiology (PAGE) consortium (http://www.pagestudy.org) was initiated in 2008 by the National Human Genome Research Institute (NHGRI) to investigate the epidemiologic architecture of well-replicated genetic variants associated with complex diseases in several large, ethnically diverse population-based studies [4]. The PAGE II consortium, cofounded by the NHGRI and the National Institute for Minority Health and Health Disparities, consists of four large, ongoing population-based studies/consortia. Three of these studies were members of the initial PAGE collaboration: the Women's Health Initiative (WHI, http://www.whi.org) [5]; the Multiethnic Cohort (MEC; http://www.uhcancercenter.org/research/the-multiethnic-cohort-study-mec) [6]; a subset of the studies comprising the consortium Causal Variants Across the Life Course (CALiCo)—Atherosclerosis Risk in Communities (ARIC, http://www.cscc.unc.edu.offcampus.lib.washington.edu/aric/) [7], Coronary Artery Risk Development in Young Adults (CARDIA; http://www.cardia.dopm.uab.edu/) [8], and the Hispanic Community Health Study/Study of Latinos (HCHS/SOL, http://www.cscc.unc.edu.offcampus.lib.washington.edu/hchs/) [9]. The fourth PAGE II study is the Charles Bronfman Institute for Personalized Medicine at the Icahn School of Medicine at Mount Sinai (ISMMS), which curates an electronic health record (EHR)-linked medical care setting biorepository (Bio*Me*^TM^, http://icahn.mssm.edu/research/institutes/ipm/programs/biome-biobank). In total, 51,650 self-reported African American/African ancestries, Hispanic/Latino, Asian/Pacific Islander, and American Indian participants have been genotyped on the Multi-Ethnic Global Array (MEGA). In comparison to previous efforts, the addition of customized content on MEGA was designed to enable deeper functional exploration of known disease-associated regions, particularly for less frequent (1–5%) and rare (<1%) genetic variation.

The MEGA array was designed predominantly through the collaborative efforts of Illumina, the Consortium on Asthma among African-ancestry Populations in the Americas (CAAPA), and PAGE II to empower GWAS in diverse ancestry populations. Customized content was included on the MEGA to improve our understanding of genetic loci associated with complex human diseases or traits and to evaluate potential genetic heterogeneity across racial/ethnic groups. Specifically, we selected custom content to: 1) explore the generalization or replication of previously-reported genotype-phenotype associations at known loci; 2) identify independent or population-specific variant associations within known associated regions; 3) leverage differences in haplotype structure (LD) across populations to hone in on likely causal variants in known trait-associated regions (‘fine-mapping’); and 4) perform candidate functional SNP analysis using variants known to be (a) clinically relevant, (b) implicated in candidate pathways, or (c) validated as regulatory through laboratory analyses such as allelic reporter assays. Given that functional variants for the majority of GWAS signals have not been identified, the design of MEGA will enable researchers to provide new understanding of the biology underlying known disease associations and improve the generalizability of risk models across populations. Here, we describe both the custom designed MEGA content and discuss valuable insights gained through design efforts that may be of interest to the greater research community.

## Methods

### MEGA content allocation

MEGA content was partitioned into two major categories: 1) ‘backbone content’ used for agnostic GWAS and exome analyses in ancestrally diverse populations, and (2) ‘PAGE hand-curated custom content’ for targeted analyses aimed at discovering causal variants. The ‘backbone’ of the array has been described elsewhere [10,11] (https://www.pagestudy.org/index.php/multi-ethnic-genotyping-array) and is summarized in Table 1. Briefly, the backbone contains highly informative SNPs for GWAS analyses in European and East Asian descent populations for backwards compatibility with other genotyping arrays. These variants, often referred to as tag SNPs, are positioned in regions of the genome with high LD and typically represent common haplotypes in populations of either European or East Asian descent. However, the vast majority of the MEGA backbone content (83%) empowers GWAS and Exome analyses in African ancestry and Hispanic/Latino populations. The backbone content also includes the tag SNP phenotype associations from the NHGRI GWAS catalog, SNPs that are mentioned in four or more publications (‘SNPs in Publications’ database from the UCSC table browser)[12], and clinically relevant variants (Table 1).

**Table 1:** Marker allocation used for design of MEGA.

Abbreviated reference	Approximate SNP allocation	Content description	Parameters informing content
***Backbone content***			
Infinium HumanCore BeadChip	250,000	Included for backwards compatibility	Highly informative GWAS tag SNPs for EUR or ASN ancestries
African Diaspora Consortium Power Chip	700,000	Augmented GWAS coverage for African ancestries	692 individuals sequenced by CAAPA, highly informative for variants with MAF>2%
Improved cross-population tagging content	300,000	Augmented GWAS coverage for diverse ancestries	New tagging strategy developed by PAGE using 1KGP Phase 3 sequencing, highly informative for variants with MAF<2%
Multiethnic exonic content	400,000	Exome markers for diverse populations	Derived from WGS/WES data from > 36,000 individuals in diverse ethnic groups, emphasizes loss of function and splice variants
NHGRI GWAS catalog	11,631	Markers (tag SNPs) from published GWAS	Includes tag SNPs not reaching genome-wide significance (p<5x10^-8^), and SNPs in high LD
SNPs in publications	5,874	SNPs listed in UCSC browser track	Mentioned by rsid number in ≥ 4 publications
Clinical and pharmacogenetic	17,000	All clinically relevant SNPs	Domain expert opinion and those annotated as deleterious
***PAGE Hand Curated Custom Content***			
Validated regulatory SNPs	2,500	Regulatory variants with *in vitro* differential function in the literature	Differential EMSA, most with differential luciferase or equivalent
Enhanced GWAS	20,000	Improved tag SNP coverage for candidate genes/regions	Minimum r^2^ of 0.8 rather than mean r^2^ of 0.6 used for backbone
Enhanced Exome	16,000	Improved exonic coverage for candidate genes/regions	All available exonic variants
Fine-mapping	16,000	Fine-mapping coverage for GWAS catalog reports	All SNPs tagged at r^2^ > 0.6 in reference population from primary GWAS report
OMIM/Clinvar ^a^	Overlaps backbone content	Clinically relevant SNPs related to traits of interest	E.g. hyperlipidemia (*LPL*, *LDLR*, etc.), BMI (*MC4R*, etc.)

^a^ OMIM/Clinvar datasets were subsequently added as back-bone content when variant classified as deleterious. Additional ‘likely deleterious’ variants were added if directly related to traits of interest; MAF = Minor Allele Frequency; 1KGP = 1000 Genomes Project; AFR, AMR, ASN, EUR = 1000 Genomes Project Phase 3 super populations; WGS/WES = whole genome sequencing/whole exome sequencing; EMSA = electrophoretic mobility shift assay

### Overview of custom content selection

The custom content available on MEGA was taken from four primary sources: large-scale GWAS, thorough literature review, publicly available variant databases (e.g. 1000 Genomes Phase 3 Project, ClinVar, OMIM, UCSC Table Browser ‘SNPs in Publications’ track), and recommendations from experts on traits of interest to PAGE II. Along with variant annotation tools (e.g. Variant Effect Predictor and ANNOVAR) these resources were used to prioritize known trait loci and select content that could be used to: 1) replicate or generalize index GWAS associations, 2) augment GWAS tagging SNPs in priority regions 3) enhance exome content in priority regions, 4) fine-map GWAS loci, (5) identify functional regulatory variants, (6) explore penetrance/frequency of clinically reported variants in a population-based cohort, and (7) identify novel variant loci in suspected candidate pathways.

### Prioritizing known GWAS trait loci

Summary statistics for the largest available GWAS were mined to nominate both known and candidate loci tagging effects for traits related to type 2 diabetes (T2D), inflammation, lipids, coronary heart disease (CHD), blood pressure, kidney disease, anthropometric measurements, various lifestyle traits like alcohol consumption, smoking, and reproductive traits. These traits were prioritized based on the availability of phenotypic data in the samples selected for genotyping in PAGE II. For GWAS-based datasets, we queried summary level data for the aforementioned traits, including both published and unpublished datasets. Using the NHGRI GWAS catalog [13] (available at: http://www.ebi.ac.uk/gwas date of access: March 19, 2014), we first identified traits directly or indirectly related to prioritized PAGE II traits. In order to maximize power, associations directly related to our traits were rank ordered and studies with smaller discovery populations were prioritized to give greater weight to variants with larger effects. The top 500 ranked associations were selected for locus refinement. For GWAS augmentation (described below), loci were defined as 100kb upstream and downstream (200kb total) of the index SNP position in the GRCh37/hg19 build.

### Variant annotation

All variants selected for inclusion were annotated using the ‘Snp 142 common’ and ‘All SNPs (142)’ datasets in the UCSC Table Browser. We also used the bedtools utility ‘intersect’ to identify overlap between variant lists, such as the list of functional SNPs, and PAGE II trait loci of interest (GWAS loci and prioritized trait group genes). Coding variants were annotated using multiple annotation methods (ANNOVAR, Variant Effect Predictor, Variant Annotation Integrator, and Variant Annotator-GATK) and the most likely deleterious annotation was assigned.

### Augmentation of GWAS tagging SNPs in priority loci

MEGA contains a GWAS scaffold designed for enhanced imputation accuracy across multiple populations [11]. In addition to providing an essential tool that can be used for more comprehensive genetic studies in diverse populations (for both less frequent and common variants), we aimed to enhance genome-wide coverage in regions previously implicated for PAGE II traits of interest. Our methodology to develop the improved multi-ethnic tag SNP selection for MEGA [14] was extended to the custom content regions. Assigning the rest of MEGA as fixed content, tags were selected to enhance coverage in the regions of interest across all 6 continental populations found in 1000 Genomes Project Phase 3 data: Admixed African-Descent (AAC), African (AFR), Americas (AMR), East Asian (ASN), European (EUR), and South Asian (SAS). Tags were selected using a minimum MAF of 1% and a minimum LD r^2^ of 0.2. GWAS loci were defined as 100kb upstream and downstream (200kb total) of the index SNP position in the GRCh37/hg19 build.

### Augmentation exome SNPs in priority loci

In the prioritized genes of interest, we lowered the MAF threshold compared to the rest of the MEGA backbone to allow inclusion of doubleton or singleton observations. Synonymous variants were also included in these genes because evidence suggests these can also be functional, whether as miRNA targets or as exonic splice enhancers/silencers [15–23].

### Fine-mapping SNPs in priority loci

For our list of GWAS catalog prioritized trait associations (see details above), we defined the variants at a locus as the index variant and all correlated SNPs with an r^2^ ≥0.6 within 200 Kb. LD calculations were derived from 1000 Genomes Phase 3 Project SNP coverage (ftp://ftp.1000genomes.ebi.ac.uk/vol1/ftp/release/20130502/ALL.chr10.phase3_shapeit2_mvncall_integrated_v2.20130502.genotypes.vcf.gz). Using vcftools v0.1.12a, pairwise LD was calculated in the relevant 1000 Genomes Project super populations: EUR was used when the index discovery sample included European-descent populations, and ASN was used for discovery populations of East or South Asian-descent [24]. Tagged SNPs were excluded if they were triallelic or they had a MAF < 1%.

### Identification of functional regulatory variants

In order to perform functionally informed analyses, we included variants that regulate gene expression as determined through laboratory analyses such as luciferase reporter assays and electromobility shift assays (EMSA). To achieve this, we mined both PubMed and Google Scholar to extract terms in the abstract and text. Our search included the following terms: “reporter”, “EMSA”, “luciferase”, “catalase”, “GFP”, or “Chromatin Immunoprecipitation.”

One of the largest challenges in this endeavor was identifying which variant was being reported. There are many different ways to reference a polymorphism, and prior to GWAS, the variant was almost never referred to by the now-standardized dbSNP rsID number. For instance, pharmacogenetic variants were often reported using a ‘gene asterisk variant’ notation (eg. CYP2A6*9) and many other studies reported variants relative to Transcription Start Sites (TSS; eg. -1031T/C). Furthermore, upstream and downstream references were arbitrarily defined as ‘+’ or ‘-‘.When a sequence was provided we used the “BLAT” tool from UCSC Genome Browser (http://genome.ucsc.edu/) to identify the correct variant. If no sequence was available but primers were provided we used the UCSC ‘In-Silico PCR’ tool. Occasionally we were able to find linkage between the rsID number and the alias in a different article using the ‘SNPs in Pubs’ track. Articles were further reviewed in an effort to verify that reports of ‘differential allelic expression’ were statistically significant after Bonferroni correction when multiple variants were analyzed.

## Results

### Augmented GWAS loci and trait related genes

After hand curation of the GWAS catalog tag SNPs directly related to the following traits were selected: T2D (n = 95), inflammation (n = 527), lipids (n = 379), CHD (n = 280), blood pressure (n = 121), kidney disease (n = 92), anthropometric (n = 644), and lifestyle or menstrual related traits (n = 107). In total, we rank ordered the list of 4,453 unique locus tag SNPs. The top 500 ranked associations were selected for locus refinement.

In addition to prioritizing these known and well-powered GWAS loci, 166 genes (Table 2) were prioritized across these eight trait groups based on thorough literature review, clinical relevance, and recommendation by experts with proficient genetic knowledge and clinical knowledge on the priority PAGE II traits. Gene coordinates were defined using the UCSC Table Browser UCSC Genes (GRCh37/hg19) and taking the union of the reported transcripts. These 166 gene coordinates were used to augment exome content and select GWAS tag SNPs.

**Table 2:** List of Prioritized Genes by Trait.

Traits	Genes of Interest
**Obesity/ Anthropometry**	*ACVR2B*, *CAPN3*, *FTO*, *GALNT10*, *IRX3*, *KSR2*, LEPR*, *MRAP2*, *MTCH2*, *NEGR1*, PCSK1*, *PRKCH*, *RMST*, *RPGRIP1L*, *SEC16B*, *SIM1*, *TFAP2B*, *TUB*,
**Kidney disease/ Blood pressure**	*ABCG2*, *APOL1*, *ARID5B*, *AS3MT*, *C1GALT1*, *C1GALT1C1*, *CUBN*, *GALNT2*, *GALNT14*, *HECW1*, *HOXA3*, *KCNK3*, *MYH9*, *PDZK1*, *RAPH1*, *SGK1*, *SLC17A1*, *SLC2A9*, *SLC22A11*, *SLC22A12*, *SLC16A9*, *ST6GALNAC2*, *TMEM171*, *UMOD*
**Coronary Heart Disease**	*CAV1/CAV2*, *KCNH2*, *NKX2-NKX5*, *MEIS1*, *PRKCA*, *SCN5A/SCN10A*, *SOX5*, *TBX5/TBX3*, *WNT11*, *ZFPM2/FOG2*
**Inflammation**	*ABO*, *ADAMTS13*, *AGER*, *APOE*, *CRP*, CD36*, *DARC*, *EPO*, *F7*, *FGB*, *FGG*, *G6PD*, *HNF1A*, *HNF4A*, *HBB*, *HFE*, *ITGB3*, *IL6R*, *JAK2*, *LCT*, LEPR*, *MPL*, *SH2B3*, *SORT1*, *TRF2*, *TUBB1*, *ZFP36L1*
**Alcohol use**	*GABRA2*, *OPRM1*, *KCNJ6*, *CHRM2*, *TAS2R38*, *TAS2R16*, *ADH1B*, *ADH1C*, *AUTS2*, *ALDH2*
**Smoking**	*CHRNA5*, *CHRNB4*, *CHRNB3*, *CHRNA6*, *CHRNA3*, *CHRNA4*, *GPR51*, *UGT2B10*
**Age at Menarche**	*ZNF483*, *FLRT2*, *CYP19A1*, PIK3R1*, *DLGAP2*, *AKT3*, *CENPW*, *LIN28B*, *LRP1B*, *RORA*
**Age at Menopause**	*ASH2L*, *HELQ*, *INHBA*, *NFAT5*, *NLRP11*, *OLFM2*, *POLG*, *PRRC2A*, *RHBDL2*, *TLK1*, *U1MC1*
**Lipids**	*AGPAT2*, *BAAT*, CD36*, *CPT1A*, *CPT2*, *CYP27A1*, *FABP2*, *HMGCR*, *LIPA*, *LMF1*, *NPC1L1*, PCSK1*, *PCSK7*, *PCSK9*, *PNPLA2*, *PPARA*, *PPARG*, *SLCO1B1*, *TM6SF2*
**Type 2 Diabetes**	*ABO*, *ACP2*, *APOL2*, *ANKH*, *ARAP1*, *COBLL1*, *CTNNAL1*, *CYB5A*, *DNLZ*, *DYRK1B*, *FAM206A*, *G6PC2*, *GLP1R*, *GPSM1*, *GRB14*, *HMGA1*, *IKBAP*, *KANK1*, *LIMK2*, *MADD*, *NUDT3*, *OR2AK2*, *PAM*, PIK3R1*, *RNF10*, *RREB1*, *SLC2A2*, *SLC30A8*, *SGSM2*, *SHBG*, *SSR1*, *TBC1D30*, *TET2*

* Genes present in multiple trait groups

### Custom content on MEGA

**Fig 1:**
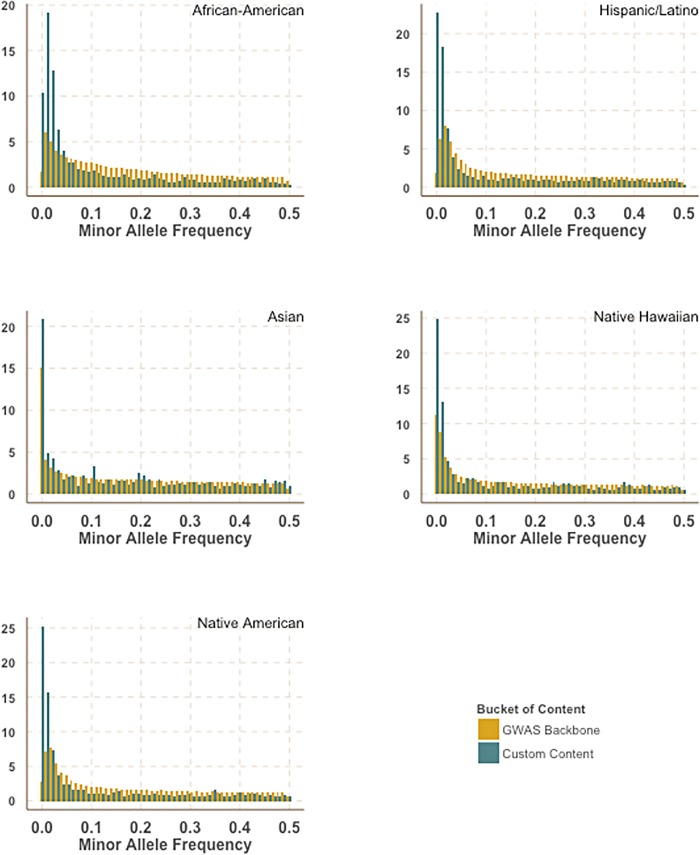
Enrichment of rarer variation in custom content. Comparison of minor allele frequency distribution between the MEGA GWAS backbone and the custom content stratified by race. Allele frequencies were calculated in PAGE II study populations.

After applying Illumina design filters to select SNPs that are less likely to fail and removing overlap between categories a total of 48,091 SNPs were included as custom design for MEGA (Table 3). In comparison to the GWAS backbone we observed that the customized content was enriched for rarer variation (Fig 1).

**Table 3:** Custom Content Variants Before and After Design.

Source	Before Design Filter	Content Included On MEGA
**SNPs in more than 4 publications**	2,894	2,849
**GWAS catalog**	4,915	4,496
**Gene-Environment Interaction**	139	122
**Pharmacogenetic**	1,616	1,495
**Unpublished GWAS**	327	215
**Enriched GWAS (S1 Table)**	28,465	25,388
**Enriched Exome (S2 Table)**	14,001	5,733
**Fine-map (S3 Table)**	15,049	11,974
**Regulatory SNPs (literature review of differential allelic assay experiments) (S4 Table)**	2,617	1,577
**Priority SNPs Requested by Trait group (S5 Table)**	3,752	2,278
**Targeted genotyping array results (S6 Table)**	129	119

### Replication or generalization of index associations

All published genome-wide significant variants related to PAGE II traits of interest were specifically chosen for inclusion on MEGA. In addition, we included 302 unpublished variants reaching genome-wide significance in PAGE I studies or in the large consortia―GIANT (https://www.broadinstitute.org/collaboration/giant/index.php/GIANT_consortium), and MAGIC (http://www.magicinvestigators.org/). These loci were reported in European, Hispanic, Asian, or African American populations. In addition, we performed a literature search in PubMed for targeted genotyping arrays (‘MetaboChip’, ‘ExomeChip’, ‘OncoArray’, and ‘ImmunoChip’) to identify additional associations that may not have been included in the NHGRI GWAS catalog. We identified an additional 102 variants significantly associated with one or more PAGE II prioritized traits.

### Augmented GWAS Tagging SNPs in Priority Regions

A total of 28,465 tag SNPs were selected for augmenting coverage over 84.7 Mb containing 157 priority genes (+/- 50kb flanking regions) and 459 highly ranked GWAS loci (+/- 100kb flanking regions). Of those tag SNPs 25,388 made it onto MEGA after QC (S1 Table). In addition to the background GWAS scaffold, there were on average 41 tag SNPs per prioritized genes and GWAS top hit. (S7 and S8 Tables)

**Fig 2:**
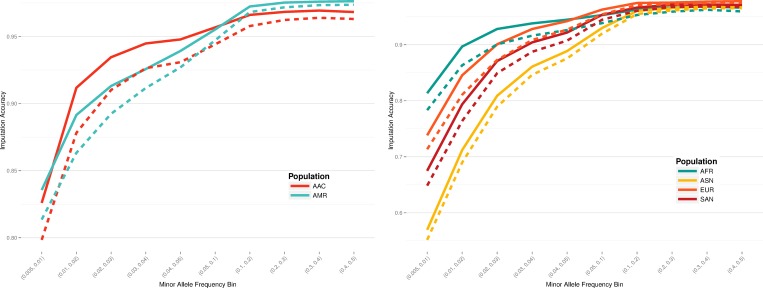
Improved imputation accuracy found with custom content sites in regions of interest. Solid lines denote the imputation accuracy of MEGA including the custom content, while dashed lines indicate the performance of MEGA without the custom content. Admixed populations are on the left, with continental populations found on the right.

Imputation accuracy was assessed through a leave-one-out internal validation approach, using the 1000 Genomes Project phase 3 data. Pearson’s correlation was estimated between the imputed dosages and the genotypes found in the original data. A scaffold of all MEGA sites was compared to MEGA excluding all custom content, including enhanced exome content, and SNPs added to fine-map previously implicated GWAS loci. An increase in performance was seen across all populations (Fig 2). The largest increase in imputation accuracy was found in low frequency loci, with a MAF below 5%. Imputation accuracy improvement was most notable for AA populations, with an accuracy increase of 3.02% points in AFR for loci with MAF between 0.5% and 1% (Table 4).

**Table 4:** Enhanced Imputation Accuracy with Custom Content Addition.

Population	Minor Allele Frequency Threshold		
	*0*.*5–1%*	*1–5%*	*>5%*
AAC	2.75%	2.63%	0.84%
AFR	3.02%	2.79%	0.98%
ASN	1.79%	1.89%	0.54%
AMR	2.20%	2.27%	0.43%
SAS	2.65%	2.38%	0.52%
EUR	2.48%	2.75%	0.43%

### Enhanced exome in priority regions

In the 166 genes of interest we selected 14,000 additional variants for inclusion on MEGA. In total 5,733 additional exome content SNPs were included on MEGA after exclusion of those with poor quality, mapping issues, or other synthesis issues. Using multiple annotation methods (ANNOVAR, Variant Effect Predictor, Variant Annotation Integrator, and Variant Annotator-GATK) and taking the most likely deleterious annotation assigned to each variant, we obtained: 36 stop gained, 2 stop lost, 97 frameshift, 49 in frame variants, 12 splicing variants, 1,144 3’ UTR, 810 5’ UTR, 1,725 non-synonymous and, 3,144 synonymous variants. The median allele frequency of these variants across populations was 0.05% (interquartile range = 0.04%-0.1%).

### Fine-mapping GWAS loci

For our list of GWAS catalog prioritized trait associations, we defined the variants at a locus as the index variant and all correlated SNPs with an r^2^ ≥0.6 within 200 Kb. This resulted in 15,049 variants representing 459 prioritized loci directly related to our traits of interest (S3 Table). We included up to 338 variants per fine-mapped locus, with an average of 33 (SD = 38) variants. After applying the Illumina design score filter of >0.5, 12,199 SNPs in 451 independent loci remained. The average number of variants per locus was similar with a mean of 27 (SD = 32) variants. The average minor allele frequency was 39% across the 12,199 variants.

### Identification of Functional Regulatory Variants

**Fig 3:**
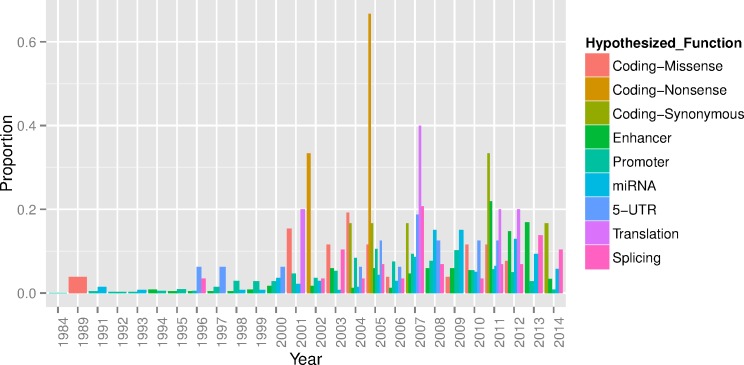
The functional hypothesis tested (‘Hypothesized Function”) by year for 2,610 variants reported in a functional allelic assay found through literature review.

In total, our literature search identified 2,610 variants showing significant differential allelic gene expression. We found that of the variants tested in an allelic assay that were also described as having function only 260 (10%) were strongly tagged (r^2^>0.6) by a GWAS index SNP. Although these variants could be the underlying functional variant for the association, it should be noted that they are not necessarily the variant underlying the association and in some circumstances there may be multiple or ethnic specific variants in a known locus. However, this supports the assertion that for the vast majority of associated loci the underlying causal variants have not been fine-mapped. Furthermore, while many of the GWAS loci identified to date are positioned in intergenic regions, until recently most of the regulatory variant follow-up has been conducted in promoters where the target gene is known (Fig 3).

### Selection of medically important variants

Putative risk variants identified by exome sequencing of familial and population based samples, as well as those derived from literature review for highly penetrant diseases related to our more common traits of interest, were also included on the array. To accomplish this, we performed a systematic literature and database search for all mutations known to cause medical traits like hyper/hypolipidemia, hypercholesterolemia, dyslipidemia, lipodystrophy, arteriosclerotic heart disease, chronic kidney disease, extreme obesity, maturity onset diabetes of the young (MODY), long QT syndrome, Brugada syndrome, alcohol and nicotine dependence or sensitivity, systemic lupus erythematosus, and airway hyper-reactivity. Using ClinVar [25], OMIM [26] and NextBio (http://www.illumina.com/informatics/research/biological-data-interpretation/nextbio.html) we obtained 2,655 candidate variants.

### Identification of variants in candidate pathways

We selected 1,546 variants for candidate pathway analyses based on their involvement with pharmacokinetics or pharmacology (for example: absorption, distribution, metabolism and excretion-ADME; or drug-metabolizing enzymes and transporters-DMET) from publicly available resources (www.pharmaadme.org, http://www.snpedia.com/index.php/Pharma_DMET, http://www.drugbank.ca/genobrowse/snp-adr).

## Discussion

The design of MEGA enables the most comprehensive examination of genetic architecture across ancestrally diverse populations to date, and thus provides a key tool for discovering important insights for many complex diseases and traits. In this manuscript, we have outlined the custom content that was hand-curated by PAGE II in order to improve our understanding of genetic loci associated with complex human diseases or traits and to evaluate potential genetic heterogeneity across racial/ethnic groups. This high-value content includes: (1) variants relevant to common, complex phenotypes of interest to PAGE II; (2) candidate functional variants in non-coding regions curated from the literature; (3) fine-mapping content selected to refine established GWAS signals reported in the GWAS catalog; and (4) augmented coverage in candidate regions containing either genes of interest or relevant GWAS associations.

The extensive effort taken to utilize existing knowledge on the genetic etiology of complex traits has enabled PAGE II to enrich the genotyping content in those regions most likely to influence our traits of interest across all populations. Ultimately, the usage of MEGA in this large multi-ethnic study will provide necessary insight into the genetics of complex diseases and help ensure that the benefits gained from genetic research are equitably distributed across diverse populations. For instance, development of genetic risk models based on European genetic architecture alone may in some cases reduce predictive accuracy in other ethnicities [27–30]. Additionally, by leveraging differences in genetic architecture across ancestral populations, transethnic studies can be used to hone in on likely functional variants in known disease or trait loci. Genetic risk models built on tagging variation that is informative across populations will ensure that all ethnic/racial groups are benefitting from the knowledge gained from the public investment in genetic research. Furthermore, by enabling functional insight in genetic risk loci, inferences on the underlying biology of complex diseases can better inform the development of treatment therapies.

The customized content selection sought to identify a set of truly functional variants from thorough literature review. MEGA includes variants that mark associations with gene expression (6689 genomic loci regulating mRNA expression-eQTLS) and those predicted in silico to have regulatory potential (RegulomeDB); these variants represent a gold standard for establishing regulatory function. The inclusion of variants that have been shown to influence gene expression in the laboratory will assist in fine-mapping associated loci and enable candidate variant associations. In our hand-curation process, we found that although more than a thousand allelic functional assays have been published, many were from before the time of GWAS and most were not conducted as a follow-up to a GWAS study. Furthermore, most of the associated loci to date did not overlap with a variant shown to be functional. Similarly, the vast majority of variants shown to be functional through laboratory assays have been positioned in the promoter, although most associations to date have been positioned in enhancer regions. As such, for the vast majority of associated loci there remains a significant amount of work to be done in identifying the underlying causal variant(s) and target genes. As efforts were taken to include variants predicted to have regulatory function or a deleterious effect in the coding region, we believe the inclusion of tagging variants that are most informative across populations will enable better prioritization of likely causal variant associations and thus streamline laboratory follow-up.

To facilitate rapid dissemination of results and methods, as well as promote new collaborations with other studies, PAGE II investigators have created a link within the study website (http://pagestudy.org/) to report usage of MEGA.

## Supporting Information

S1 Table Variants selected for enriching GWAS coverage. (TXT)

S2 Table Variants selected for enriching exome coverage. (TXT)

S3 Table Variants selected for enriching locus fine-mapping content. (TXT)

S4 Table Variants included from regulatory literature review. (TXT)

S5 Table Variant prioritized by trait groups. (TXT)

S6 Table Targeted genotyping results. (TXT)

S7 Table Number of GWAS tagging SNPs per gene. (TXT)

S8 Table Number of GWAS tagging SNPs per SNP. (TXT)

## Abbreviations

AAC: Admixed African-Descent 1000 Genomes Phase 3 super population code
ADME: Absorption, Distribution, Metabolism, Excretion
ARIC: Atherosclerosis Risk in Communities
AFR: African 1000 Genomes Phase 3 super population code
AMR: Ad Mixed American 1000 Genomes Phase 3 population
ASN: East Asian 1000 Genomes Phase 3 population
CALiCo: Causal Variants Across the Life Course
CARDIA: Coronary Artery Risk Development in Young Adults
CHD: Coronary Heart Disease
dbGaP: Database of Genotypes and Phenotypes
DMET: Drug-Metabolizing Enzymes and Transporters
EMSA: Electrophoretic Mobility Shift Assay
EUR: European 1000 Genomes Phase 3 super population code
GATK: Genome Analysis Toolkit
GIANT: Genetic Investigation of Anthropometric Traits consortium
GWAS: genome-wide association study(ies)
HCHS/SOL: Hispanic Community Health Study/ Study of Latinos
ISMMS: Icahn School of Medicine at Mount Sinai
LD: Linkage Disequilibrium
MEC: Multiethnic Cohort Study
MEGA: Multi-Ethnic Genotyping Array
miRNA: microRNA
MODY: Maturity Onset Diabetes of the Young
NHANES: National Health and Nutrition Examination Surveys
NHGRI: National Human Genome Research Institute
NIH: National Institutes of Health
OMIM: Online
PCR: Polymerase Chain Reaction
PAGE II: Population Architecture using Genetics and Epidemiology Phase II
SAS: South Asian 1000 Genomes Phase 3 super population
SNP: Single Nucleotide Polymorphism
T2D: Type 2 Diabetes
Tag SNP: tagging SNP (also referred to as index SNP) utilized in GWAS
UCSC: University of California Southern California
UTR: Untranslated Region
WHI: Women’s Health Initiative
